# The *TCO* concept in German forensic homicide offenders with schizophrenia spectrum disorders – new findings from a file-based, retrospective cross-sectional study

**DOI:** 10.3389/fpsyt.2024.1404263

**Published:** 2024-06-11

**Authors:** Hannelore Findeis, Maria Strauß, Hans-Ludwig Kröber

**Affiliations:** ^1^ Klinik für Psychiatrie und Psychotherapie, Universitätsklinikum Leipzig, Leipzig, Germany; ^2^ Institut für Forensische Psychiatrie, Charité Berlin, Berlin, Germany

**Keywords:** TCO, homicide, schizophrenia, psychopathology, forensic psychiatry

## Abstract

**Introduction:**

There is evidence that there is a small group of people with schizophrenia spectrum disorders who are more likely to commit homicide than those in the general population. However, there is limited knowledge about the psychopathology that leads to homicide in this group. The aim of this study was to examine two commonly used definitions of the *Threat/Control-Override (TCO)* concept, which aims to identify a certain risk of serious violence in patients with schizophrenia spectrum disorders.

**Methods:**

This is a sub analysis of a file-based, retrospective and exploratory cross-sectional study. All forensic homicide offenders with schizophrenia spectrum disorders who were detained at the Forensic Hospital Berlin as of 31 December 2014 were examined for the occurrence of *TCO* according to two commonly used definitions.

**Results:**

Of a total of 419 forensic patients with schizophrenia spectrum disorders, 78 committed homicide (18.6%). The forensic homicide offenders with schizophrenia spectrum disorders were characterised by being male, unemployed, single and having committed (attempted) manslaughter. Irrespective of the definition used, the entire *TCO* complex was present in less than a third of the sample. In both definitions, *Threat* symptoms were slightly less frequent than *Control-Override* symptoms. While *Threat* symptoms occurred less frequently in Stompe et al.’s definition, *Control-Override* symptoms were the most common. With regard to Kröber’s definition of *Threat* and *Control-Override*, the situation is exactly the opposite.

**Discussion:**

Regarding the entire *TCO* complex, Kröber’s definition seems a little more open and Stompe et al.’s more strict (38.5% *vs*. 35.9%). Since *TCO* only occurs in about one third of the subjects in both definitions, neither definition appears to be conclusive. A combination with proportions from both definitions could be a contribution to a future definition of *TCO*. The present study provides scarcely published primary data on psychopathology in homicide offenders with schizophrenia spectrum disorders, especially on the much discussed *TCO* concept in two definitions. In order to determine the most useful definition of *TCO*, to avoid false positives and to identify clear psychopathological risk symptoms, larger samples and comparative studies with offenders and non-offenders should be conducted in the future.

## 1 Introduction

The global prevalence of schizophrenic psychosis is estimated to be 0.5 – 1%. In Germany, 0.5% of patients with schizophrenia spectrum disorders are admitted to a forensic psychiatric hospital according to § 63 of the German Criminal Code (Strafgesetzbuch, StGB). This represents a relatively small group of offenders, and the majority of people with schizophrenia spectrum disorders will never commit a violent crime in their lives (1). These patients are more likely to become victims of a violent crime (2). Nevertheless, several studies have shown a moderately significant association between schizophrenia and violence (3–8; and many others), especially with serious violent crime (5, 9) and homicide (10–14). These findings were replicated numerous times in various study designs, samples and social systems (15).

It is believed that about 5 – 20% of all homicide offences are being committed by patients with schizophrenia spectrum disorders (16–18).

According to the German Criminal Code, homicide is defined as a crime against human life, such as murder, manslaughter, involuntary manslaughter, induced abortion, homicide on demand and attempts to commit these crimes. Only attempted and completed murder and manslaughter were committed by the subjects of this research.

There is evidence that the paranoid subtype is the most common in violent patients with schizophrenia spectrum disorders (4). According to Schanda et al. (13), this subtype is present in 63.4% of the male and 47.1% of the female homicide offenders with schizophrenia spectrum disorders. Moreover, there is evidence that the delusional misidentification of people is associated with a higher risk of committing serious violent crime (9, 19–21). According to this, Prüter (22) found that 80% of the serious violent offences of patients with schizophrenia spectrum disorders were directly associated with their individual delusional content. This finding was confirmed by the meta-analysis and systematic review of Witt et al. (7) and the retrospective study of Swanson et al. (23).

In addition to that, comorbid personality disorders (7, 24) and substance abuse are associated with a significant higher risk of committing serious violent crime (7, 25).

In order to bring light into the nature of the serious violent and homicidal behaviour of patients with schizophrenia spectrum disorders, several authors classified the offenders and offences depending on different characteristics (26 and 27 with regards to the nature of aggression that leads to the homicide offence; 1, 28 with regards to three different types of violent offenders with schizophrenia spectrum disorders depending on their course of disease; 29 with regards to three types of offences depending on the association of psychotic symptoms, the constellation of the homicide offence and the degree of violence that leads to the homicide; 30 with regards to two different types of offenders with schizophrenia spectrum disorders depending on their development of delinquency).

As a result of their retrospective study (*n* = 732), Link and Stueve (31) formulated a psychopathological syndrome which they called *Threat/Control-Override* (*TCO*) as another form of classification of homicidal behaviour and homicidal offences in patients with schizophrenia spectrum disorders. It is a psychopathological constellation that places patients with schizophrenia spectrum disorders at particular risk of committing a violent crime. *Threat* was defined as feeling threatened by radiation or body hallucinations. *Control-Override* was defined as feeling controlled by an external force through thought withdrawal or thought insertion, and being at the mercy of something evil with depersonalisation and derealisation. Earlier, in the 1980s, Taylor (9) described similar findings in which schizophrenic violent criminals were characterised by a degree of florid psychotic psychopathology.

The statistically significant relationship between *TCO* and a higher risk of committing violent crimes among patients with schizophrenia spectrum disorders has been replicated in several studies (32–37). Swanson et al. (35) showed that patients with *TCO* had a twofold risk of committing violent crimes compared to psychotic patients without *TCO* and a fivefold risk compared to the general population. Later, Link et al. (32) showed that both *Threat* and *Control-Override* symptoms were independently associated with violent behaviour in patients with schizophrenia spectrum disorders.

On the other hand, there has been early criticism of *TCO* studies. Mullen (38) criticised the authors of *TCO* studies for producing too many false positives (*TCO* among non-offenders) and not discussing them sufficiently. In addition, Appelbaum et al. (39), using data from the MacArthur Violence Risk Assessment Study, found that the apparently statistically significant relationship between *TCO* and violent behaviour in patients with schizophrenia spectrum disorders disappeared when “anger” and “impulsivity” were included as covariates. Instead, they found that violent behaviour was associated with the absence of *Control-Override* symptoms.

Using data from the MacArthur Violence Risk Assessment Study, Teasdale et al. (40) found that *TCO* was not suitable for predicting violent behaviour in women with schizophrenia spectrum disorders. Rather, they showed that *TCO* was statistically significantly associated with less violent behaviour in women with schizophrenia spectrum disorders. One possible explanation for this is that men and women react differently to (delusional) threats: While males react according to “fight-or-flight”, females tend to react according to “tend-and-befriend”, which implies less violent behaviour. For males, Teasdale et al. (40) found neither a positive nor a negative relationship between violence and the full *TCO* concept. However, the authors did find an isolated statistically significant relationship between *Threat* symptoms and violent behaviour (40).

Kröber and Lau (41) concluded that validation of the *TCO* concept in larger samples is still needed.

The retrospective comparative study by Stompe et al. (14, 42) with delinquent and non-delinquent subjects with schizophrenia spectrum disorders showed no statistical significance regarding the prevalence of *TCO* in the two groups. However, when comparing three groups of subjects with schizophrenia spectrum disorders – serious violent offenders, moderate violent offenders and non-offenders **–** there was a statistically significant association between *TCO* and the serious violent offender group. However, the lifetime prevalence of *TCO* symptoms was rather high in all groups (serious violent offenders 97.1%; non-offenders 90.5%; moderate violent offenders 76.6%). The accumulation of *TCO* in the serious violent offender group was mainly attributed to *Threat* symptoms. These were defined as a specific form of persecutory delusion in which the patient believes that his or her life is in acute danger. The prevalence of *Threat* symptoms was 70.7% in serious violent offenders, 16.7% in moderate violent offenders and 46.1% in non-offenders. *Control-Override* symptoms were not associated with a higher risk of violent behaviour (14, 42). Nederlof et al. (43) confirmed these findings in a multicentre cross-sectional study: The statistically significant association between *TCO* and violent behaviour was solely due to *Threat* symptoms. In addition, they found that the variables “anger” and “anxiety” were also statistically significantly associated with violent behaviour.

Witt et al. (7) did not find a statistically significant relationship between *TCO* and violent behaviour. However, it should be noted that the authors of this study counted aggressive verbal behaviour as violent behaviour, whereas the original definition only included physical violence (31).

The contradictory findings regarding *TCO* and serious violent behaviour and crime could be a result of different study designs, samples, survey methods and definitions of general terms such as violence, delinquency, psychosis and *TCO* itself (44). In addition, most authors use three to four criteria to define *TCO*, which may lead to an underreporting of relevant psychopathological symptoms (43). The most common definitions of *TCO* in the literature are summarised in **Table 1**.

**Table 1:** TCO definitions.

Authors	* TCO* definition
Link and Stueve (31)	1. Thought/mind control 2. Thought insertion 3. Delusional belief that other people wish to harm
Swanson et al. (35)	1. Thought/movement control 2. Thought insertion/withdrawal 3. Delusional belief that other people are plotting/hurting/poisoning 4. Delusional belief that other people are following
Link et al. (32)	1. Thought/mind control 2. Thought insertion 3. Delusional belief that other people wish to harm 4. Persecutory delusions 5. Delusions of control
Appelbaum et al. (39)	1. Delusional belief of being under external control 2. Thought insertion 3. Thought withdrawal 4. Delusional belief of being hypnotized/under magic 5. Delusional belief of being spied 6. Delusional belief that other people are following 7. Delusional belief of being part of secret experiments 8. Delusional belief of plotting/hurting/poisoning
Stompe et al. (42)	1. Persecutory delusion and/or delusion of poisoning or other delusional beliefs of being life threated 2. Thought withdrawal 3. Thought insertion 4. Delusional belief of being under external control of emotions, actions and desires
Kröber (1)	1. Immediate subjective experience of threat (persecution, radiation, poisoning) 2. Being under control of external power (Thought withdrawal/influence) 3. Being on someones mercy and initiating overwhelm (loss of self, destruction)
Nederlof et al. (43), *TCOQ*	1. Other people have tried to poison me or to do me harm. 2. Someone has deliberately tried to make me ill. 3. Other people have been secretly plotting to ruin me. 4. Someone has had evil intentions against me. 5. I have the thought that I was being followed for a special reason. 6. People have tried to drive me insane. 7. I am under the control of an external force that determines my actions. 8. Other people control my way of movements. 9. Other people can insert thoughts into my head. 10. My thoughts are dominated by an external force. 11. I have the feeling that other people can determine my thoughts. 12. Other people can insert thoughts into my mind. 13. I have the feeling that other people have control over me. 14. My life is being determined by something or someone except for myself.
Stompe (44)	1. Systematic delusion of persecution or poisoning concomitant with massive death threat by particular people or groups of people 2. Diffuse feeling of threat (of being tapped/observed) 3. Thought withdrawal/insertion, hearing of thoughts and hallucinations (imperative, commentating, dialogue, coenesthesia)
Stompe et al. (14)	1. Systematic delusion of persecution or poisoning concomitant with massive death threat by particular people or groups of people 2. Thought withdrawal 3. Thought insertion 4. Delusional belief that external powers are in control of one´s own emotions, actions and desires

TCO, Threat/Control-Override; TCOQ, Threat/Control-Override Questionnaire.

In contrast to the definition of Swanson et al. (35), thought withdrawal, movement control and persecutory delusions are not part of the definition in the *TCO* concept of Link and Stueve (31). Appelbaum et al. (39) provided further information on the psychopathological symptom of movement control: The feeling of being hypnotised, trapped, spied on or followed, as well as the delusional belief of being part of secret experiments. According to Stompe (44), *Threat* is a systematised delusion of persecution or poisoning and the belief that one is in life-threatening danger from certain people. Like Swanson et al. (35), he distinguishes between persecutory delusions and delusions of poisoning. Following Kurt Schneider’s “Erstrang” symptoms, Stompe (44) describes the *Control-Override* symptoms as thought withdrawal and thought insertion, thought hearing and hallucinations (imperative, acoustic, dialogue, coenesthesia). Later, Stompe specified *Threat* as a serious persecutory delusion in which the patient is in a strong delusional belief of acute life-threatening danger (45).

Kröber (1) characterised *TCO* as a “very acute hallucinatory-paranoid syndrome” that is accompanied by an existential threat and a loss of self in a complex cognitive, emotionally aggressive situation.

Nederlof et al. (43) provided the most detailed definition of *TCO* to date with the *Threat/Control-Override Questionnaire* (*TCOQ*): the first six questions reflect *Threat* symptoms and the following eight questions reflect *Control-Override* symptoms (**Table 1**).

The *TCO* concept can be understood as a condensation of various typical but not specifically schizophrenic symptoms. According to the available literature, there seems to be a positive relationship between *Threat* and serious violent behaviour such as homicide.

A definitive definition of *TCO* is still lacking. For this reason, the present study aims to compare two established German-language definitions by studying a sample of homicide offenders with schizophrenia spectrum disorders who were detained at the Berlin Forensic Hospital under § 63 of the German Criminal Code (StGB) and to provide an appropriate definition of *TCO*.

Furthermore, a more comprehensive knowledge of the specific psychopathology of schizophrenic violence and the motivation for homicide offenders with schizophrenia spectrum disorders may not only help to identify future patients at high risk for violent behaviour, but also contribute to the destigmatisation of people with serious mental disorders (46, 47).

## 2 Material and methods

### 2.1 Study design and participants

The data in this study come exclusively from a dataset generated by the first author of this study. It is a sub analysis of a file-based, retrospective and exploratory cross-sectional study. The original study was conducted to obtain a complete overview of all forensic homicide offenders detained in the Berlin Forensic Hospital according to §§ 63 and 64 of the German Criminal Code (StGB). In this sub analysis all homicide offenders with schizophrenia spectrum disorders at the Berlin Forensic Hospital were assessed for certain sociodemographic and psychopathological variables. Diagnosis was made according to the ICD-10 (International Statistical Classification of Diseases and Related Health Problems). The sample consists exclusively of male and female subjects with schizophrenia spectrum disorders who had committed attempted or completed murder or manslaughter and who were incarcerated at the Berlin Forensic Hospital on 31 December 2014 (*N* = 78). The sample represents a total capture of the persons concerned.

### 2.2 Assessment/materials

The data were collected exclusively by the first author of the study between January 2014 and November 2015. For this purpose, the medical records of the patients from the Berlin Forensic Hospital were reviewed and both the conviction of the index offence and the forensic psychiatric examination were analysed. The medical records were reversibly pseudonymised and analysed with the approval of the Senate Administration for Justice Berlin. The protection of the data privacy of each patient was ensured according to the General Data Protection Regulation (Datenschutzgrundverordnung; (EU) 2016/679; in force since 25 May 2018). There was no personal exploration or written survey of the subjects.

The present study uses the *TCO* definitions of (14; **Table 2**) and (1; **Table 3**). Due to the lack of naming of concrete psychopathological symptoms in both definitions, an operationalisation was performed to transform the respective definitions of *Threat* and *Control-Override* into psychopathological symptoms that refer to the Manual for Assessment and Documentation of Psychopathology in Psychiatry (48).

**Table 2:** Operationalisation of TCO symptoms referring to Stompe et al. (14).

*TCO* symptoms	Definition referring to Stompe et al. (14)	Operationalisation
*Threat*	- Systematic delusion of persecution or poisoning concomitant with massive death threat by particular people or groups of people	1. Persecutory delusion or delusion of poisoning 2. Systematic delusion 3. Hostile and destructive delusion 4. Highly affective involvement in the delusion
*Control-Override*	- Thought withdrawal - Thought insertion - Delusional belief that external powers are in control of one´s own emotions, actions and desires	1. Thought withdrawal/insertion

n, sample size; TCO, Threat/Control-Override.

**Table 3:** Operationalisation of TCO symptoms referring to Kröber (1).

*TCO* symptoms	Definition referring to Kröber (1)	Operationalisation
*Threat*	- Immediate subjective experience of threat (persecution, radiation, poisoning)	1. Persecutory delusion or delusion of poisoning 2. Hostile and destructive delusion 3. Highly affective involvement in the delusion
*Control-Override*	- Being under control of external power (Thought withdrawal/influence) - Being on someones mercy and initiating overwhelm (loss of self, destruction)	1. Thought withdrawal/insertion 2. Depersonalisation/derealisation

n, sample size; TCO, Threat/Control-Override.

The definition of Stompe et al. (14) was chosen because it retrospectively compared offenders with schizophrenia spectrum disorders with moderate on the one hand and serious on the other hand, as well as with no offending at all. A statistically significant relationship with *TCO* was found only in the group of offenders subjects with schizophrenia spectrum disorders with serious crimes (14). Kröber (1) definition was chosen because of its precise description of common psychopathological symptoms. *Threat* and *Control-Override* were considered to be fulfilled if all attributed variables were consistent.

### 2.3 Analysis

The statistical analysis of the 24 variables was carried out with SPSS (IBM SPSS Statistics, for Mac, version 29.0) using descriptive statistics. Certain results are presented graphically.

## 3 Results

As of 31 December 2014, 419 patients with schizophrenia spectrum disorders were incarcerated in the Berlin Forensic Hospital. Of these, 78 were patients with attempted or completed homicide (18.6%). All were detained under § 63 of the German Criminal Code (StGB) and, with the exception of seven subjects with reduced criminal responsibility, all were found not guilty. Almost a quarter of the subjects committed (attempted) murder and more than three quarters committed (attempted) manslaughter. Sociodemographic variables are shown in **Table 4**.

**Table 4:** Sociodemographic variables (n = 78).

Variable	Value	F2x
Age at index offence, *M (SD), spread, median*		32.5 (9.6), 48.0, 29.0
Sex, *n (%)*	Male Female	69 (88.5) 9 (11.5)
Index offence, *n (%)*	Murder Attempted murder Manslaughter Attempted manslaughter	9 (11.5) 10 (12.8) 30 (38.5) 29 (37.2)
Paragraph criminal responsibility, *n (%)*	§ 20 StGB § 21 StGB	71 (91.0) 7 (9.0)
Marital status, *n (%)*	Relationship No relationship	17 (21.8) 61 (78.2)
Occupational status, *n (%)*	Unemployed Retirement pension Employed Studies/training	62 (79.5) 7 (9.0) 5 (6.4) 4 (5.1)
Living status, *n (%)*	Proprietary apartment Home Homeless	54 (69.2) 11 (14.1) 13 (16.7)
Financial status, *n (%)*	Proprietary income Receipt of benefits or pensions No income	8 (10.3) 59 (75.6) 11 (14.1) 11 (14.1)
Nationality, *n (%)*	German German with migration background Not German	45 (57.7) 9 (11.5) 24 (30.8)
Variable	Value	F2x
Graduation status*, n (%)*	No graduation Secondary school level General qualification for university entrance	20 (25.6) 47 (60.3) 11 (14.1)
Higher educational status, *n (%)*	No higher education Completed vocational education Completed studies	50 (64.1) 27 (34.6) 1 (1.3) 1 (1.3)

n, sample size; M, mean; SD, standard deviation; F2x, schizophrenia spectrum disorders; StGB, Strafgesetzbuch (German Criminal Code).

The *TCO*-defining psychopathological symptoms were as follows (**Table 5**): systematic delusions were the most common (73.1%), followed by hostile and destructive delusions (70.5%) and highly affective involvement in the delusion (69.2%). Thought withdrawal or thought insertion (56.4%) and persecutory delusions (51.3%) occurred in just over half of the homicide offenders with schizophrenia spectrum disorders. About a third of the subjects suffered from depersonalisation and derealisation (34.6%) and less than a third from delusions of poisoning (26.9%).

**Table 5:** Psychopathology at time of index offence (n = 78).

Variable	F2x
Highly affective involvement in the delusion, *n (%)*	54 (69.2)
Delusion of poisoning*, n (%)*	21 (26.9)
Systematic delusion*, n (%)*	57 (73.1)
Thought withdrawal/insertion, *n (%)*	44 (56.4)
Depersonalisation/derealisation*, n (%)*	27 (34.6)
Persecutory delusion*, n (%)*	40 (51.3)
Hostile and destructive delusion, *n (%)*	55 (70.5)

n, sample size; F2x, schizophrenia spectrum disorders.

**Figure 1:**
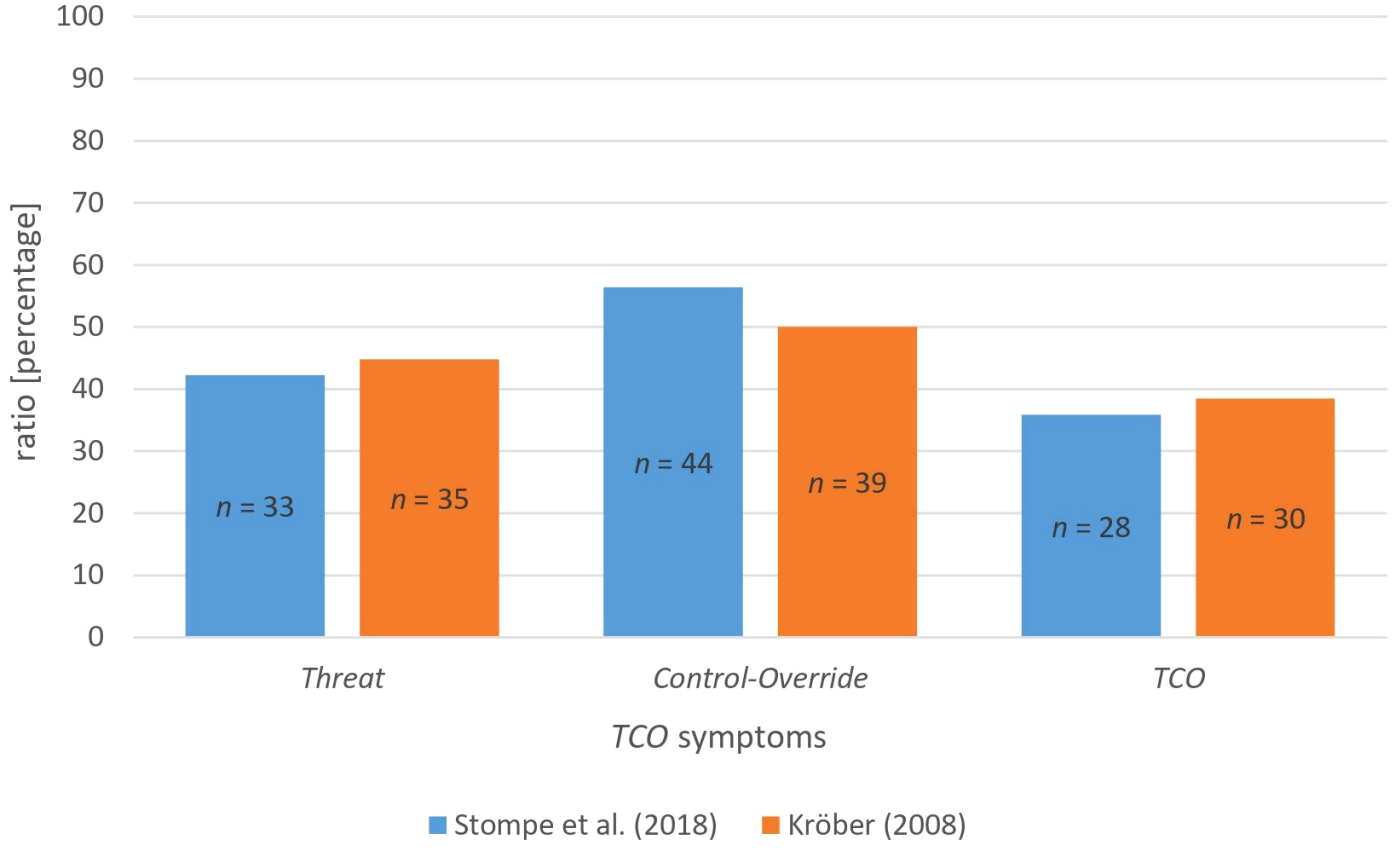
*TCO* symptoms (n = 78). Notes: n, sample size; TCO, Threat/Control-Override.

Following the definition of Stompe et al. (14), 33 subjects (42.3%) exhibited *Threat* symptoms at time of the index offence. *Control-Override* symptoms were present in 44 subjects (56.4%). The entire *TCO* complex occurred in 28 subjects (35.9%; **Figure 1**, **Table 6**). Following the definition of Kröber (1), 35 subjects (44.8%) showed *Threat* symptoms and 39 subjects (50.0%) *Control-Override* symptoms. The full *TCO* complex occurred in 30 subjects (38.5%; **Figure 1**, **Table 6**).

**Table 6:** Threat/Control-Override (n = 78).

*TCO* symptoms	Stompe et al. (14)	Kröber (1)
*Threat*, *n (%)*	33 (42.3)	35 (44.8)
*Control-Override*, *n (%)*	44 (56.4)	39 (50.0)
*TCO, n (%)*	28 (35.9)	30 (38.5)

n, sample size; TCO, Threat/Control-Override.

## 4 Discussion

The results regarding sociodemographic data are in line with the available literature (14, 25, 49–51; and many others): More than three-quarters of all subjects are male, single, unemployed and in receipt of benefits or pensions. Regarding the age at the index offence (on average 32.5 years in the present study), the findings of Schanda (13; the majority of the subjects were older than 25 years) can be confirmed. In addition, almost one in five subjects was homeless at the time of the index offence.

In terms of psychopathology at the time of the index offence, the subjects most frequently suffered from both systematic, hostile and destructive delusions and persecutory delusions. The findings are consistent with the frequently replicated findings of specific and intense psychopathology at the time of the index offence (7, 14, 22, 23, 42, 52).

With regard to the occurrence of *TCO*, the entire complex was present in just over one third of all subjects. These findings are in contrast to the data of Stompe et al. (14), who found that almost two thirds of all examined subjects who committed a serious violent offence met the criteria for the entire *TCO* complex (70.9%). Contrary to reports in the literature (where *TCO* was present mainly due to *Threat* symptoms; 14, 40, 42, 43), *Control-Override* symptoms were more frequent than *Threa*t symptoms in the present study. Furthermore, *Threat* symptoms according to the definition of Stompe et al. (14) occurred the least often, while *Control-Override* symptoms according to Stompe et al. (14) occurred the most often. An explanation for this could be the more open definition of *Control-Override* in the definition of Stompe et al. (14) and therefore more subjects fulfilling their criteria. Conversely, the precise definition of *Threat* symptoms, also compared to Kröber (1) definition, leads to fewer subjects showing these symptoms. In addition to systematic, hostile and destructive delusions and persecutory delusions, Stompe et al. (14) also include a highly affective involvement in the delusion in the definition of *Threat.* Kröber included derealisation and depersonalisation in his definition of *Control-Override*, which makes it stricter and ultimately fewer subjects exhibited *Control-Override* symptoms (50.0% *vs*. 56.4%).

The different results when comparing data from the literature and the present study could be explained as follows: Due to the lack of specific psychopathological symptoms in both definitions, an operationalisation was necessary to transform the definitions of *Threat* and *Control-Override* into AMDP-based psychopathological symptoms. This transformation may result in a lack of information on the psychopathology of homicide offenders with schizophrenia spectrum disorders at the time of the index offence and is therefore prone to error. Furthermore, it remains unclear whether Kröber (1) and Stompe et al. (14) meant their *TCO* definitions to be fulfilled when all operationalised symptoms were applicable, or whether one positive symptom was sufficient to fulfil the *Threat* or *Control-Override* criteria. In the present study, *Threat* and *Control-Override* were only counted as fulfilled when all operationalised variables were present in the subject.

*TCO* is said to represent a psychopathological symptom complex, the presence of which represents a particular risk of imminent violent offence in patients with schizophrenia spectrum disorders. As all subjects in the sample have committed a serious violent offence (homicide), *TCO* should theoretically be fulfilled for as many subjects as possible. In order to decide which of the two definitions of *TCO* examined is the most appropriate, both proved to be less sensitive in terms of recognising an imminent violent offence. The majority of subjects did not fulfil the *TCO* criteria according to the two definitions. Both the definition of Stompe et al. (14) and the definition of Kröber (1) seem to be rather inappropriate. A hypothesis is that the future application of a *TCO* definition based on the *Threat* symptoms of Kröber (1) and the *Control-Override* symptoms of Stompe et al. (14) could be useful. To avoid additional operationalisation, a future definition should capture psychopathological symptoms that are as precise and clearly explorable as possible. Furthermore the psychopathological symptoms that have to be fulfilled for both *T*- and *CO*-symptoms should be reconsidered regarding the question whether they all have to be applicable or whether two out of the three *T*-symptoms and one out of the two *CO*-symptoms are sufficient already. *TCO* could therefore be defined as follows: Two symptoms out of persecutory delusion or delusion of poisoning, hostile and destructive delusions and a highly affective involvement in the delusion and one symptom out of thought withdrawal and thought insertion.

Due to the *post-hoc* nature of the present study, the results do not allow any conclusions to be drawn about the predictive value of *TCO* for serious violent crime. The event studied (homicide) was established in the past and applied to the entire sample. The problem of the frequent false positive results described early on in the literature (14, 38, 40) can therefore not be solved with the present study. A critical examination of the *TCO* definition with regard to the avoidance of false positive results (patients with *TCO* who do not commit a violent offence) should be the subject of future studies.

There is a considerable difference in the scope of the sentences analysed in the index offence and in the forensic psychiatric examinations. It is not possible to prove whether, due to incomplete documentation, relevant information on the current subjects is missing. As the forensic psychiatric reports were written by different authorised experts and the sentences of the index offence were written by different authors, variations in diction or bias cannot be ruled out. Due to the small number of female subjects (*n* = 9), male and female subjects were analysed together in the present study. No face-to-face interviews were conducted. This could be a limitation, but at the same time the present file-based method represents a methodological strength of the study. The question of the study includes the development of the patients up to the commission of the index offence, and the forensic psychiatric examinations were carried out in the majority of cases at the time of the index offence. For this reason, the psychopathology of the patients could be better represented than by interviewing them (in most cases) many years after the index offence.

In addition, the present study represents a total survey of the sample studied. To the best of our knowledge, this study is the first to focus on a comparative investigation of the presence of *TCO* in forensic homicidal offenders with schizophrenia spectrum disorders depending on two different definitions of *TCO*.

Therefore, the present study provides little published primary data on psychopathology in forensic offenders with schizophrenia spectrum disorders, especially on the much debated *TCO* concept in two definitions. In order to identify clear psychopathological risk symptoms and to determine the most useful *TCO* definition, larger samples and comparative studies with offenders and non-offenders should be conducted in the future.

However, despite the persuasive evidence for a significant association between schizophrenia spectrum disorders and serious violent behaviour, it still remains unclear why the main part of the patients with schizophrenia spectrum disorders won´t commit any violent offence in their lives (despite of having similar psychotic symptoms; 28, 53) and therewith the final exploration of homicidal behaviour in patients with schizophrenia spectrum disorders.

## Data availability statement

The raw data supporting the conclusions of this article will be made available by the authors, without undue reservation.

## Ethics statement

The studies involving humans were approved by Senatsverwaltung für Justiz des Landes Berlin. The studies were conducted in accordance with the local legislation and institutional requirements. Written informed consent for participation was not required from the participants or the participants’ legal guardians in accordance with the national legislation and institutional requirements.

## Author contributions

HF: Writing – review & editing, Writing – original draft, Visualization, Methodology, Investigation, Funding acquisition, Formal analysis, Data curation, Conceptualization. MS: Writing – review & editing, Validation, Supervision, Resources, Funding acquisition. HK: Writing – review & editing, Validation, Supervision, Methodology, Data curation, Conceptualization.
